# *Bacteroides uniformis*-generated ornithine alleviates alcohol-associated liver disease via Sirt4-mediated mitochondrial regulation

**DOI:** 10.1080/19490976.2026.2756713

**Published:** 2026-11-08

**Authors:** Hang Shu, Mengmeng Zhang, Hang Zhou, Jingjing Ji, Panyi Hu, Qiuyu Chen, Linxi Xie, Jinxin Li, Hua Wang, Long Xu

**Affiliations:** a Department of Oncology, the First Affiliated Hospital of Anhui Medical University, Hefei, China; b School of Basic Medical Sciences, Anhui Medical University, Hefei, China; c Inflammation and Immune Mediated Diseases Laboratory of Anhui Province, Hefei, China; d Key Laboratory of Anti-inflammatory and Immune Medicine (Ministry of Education, Anhui Medical University), Hefei, China; e Innovation and Entrepreneurship Laboratory for College Students, Anhui Medical University, Hefei, China; f School of Pharmacy, Anhui Medical University, Hefei, China; g Department of Radiology, Huaxi MR Research Center (HMRRC), Institute of Radiology and Medical Imaging, West China Hospital of Sichuan University, Sichuan, China; h Department of Anesthesiology, The First Affiliated Hospital of University of Science and Technology of China, Hefei, China; i Institute of Clinical Immunology, The First Affiliated Hospital of Anhui Medical University, Hefei, China

**Keywords:** *Bacteroides uniformis*, alcohol-associated liver disease, ornithine, Sirt4, gut microbiota

## Abstract

Gut microbiota plays a pivotal role in alcohol-associated liver disease (ALD), yet the contributions of individual bacterial strains and their metabolites remain poorly defined. Here, we analyzed 16S rDNA sequencing data from an ALD patient cohort and validated the findings using the NIAAA mouse model combined with multi-omics approaches. *Bacteroides uniformis* was significantly depleted in ALD patients, and supplementation with live *B. uniformis* alleviated ALD in mice by reducing hepatic steatosis, inflammation, and oxidative stress. Metabolomic analysis identified ornithine as a key metabolite produced by *B. uniformis*, and direct ornithine administration largely recapitulated the protective effects. Mechanistically, ornithine upregulated hepatic Sirt4, thereby restoring mitochondrial homeostasis, whereas pharmacological inhibition of Sirt4 abolished these effects. Dietary flaxseed oil increased intestinal *B. uniformis* abundance and hepatic Sirt4 expression, ameliorating ALD outcomes. These findings reveal a *B. uniformis*–ornithine–Sirt4 axis that modulates ALD via mitochondrial regulation and highlight potential microbial and metabolic therapeutic targets.

## 1. Introduction

Alcohol‑associated liver disease (ALD) remains one of the leading causes of chronic liver disease, cirrhosis, and liver-related mortality worldwide.^1^ ^,^ ^2^ The pathogenesis of ALD is complex and involves multiple interconnected processes, including oxidative stress, inflammatory responses, intestinal barrier dysfunction, and mitochondrial injury.^2^ Among these mechanisms, disruption of the gut–liver axis has emerged as a central driver of disease progression. Chronic alcohol consumption induces profound alterations in gut microbial composition and function, resulting in the loss of beneficial commensals, expansion of pathobionts, and dysregulated production of microbiota-derived metabolites.^3^ ^,^ ^4^ These changes contribute to intestinal barrier breakdown, systemic inflammation, and hepatic injury. Consequently, identifying beneficial gut microbes and elucidating the molecular mechanisms through which they communicate with the host have become important goals in ALD research.

*Bacteroides uniformis* (*B. uniformis*) is a common commensal bacterium that is highly abundant in the healthy human intestine and has attracted increasing attention because of its metabolic and immunomodulatory properties. Previous studies have demonstrated that *B. uniformis* improves glucose homeostasis and metabolic dysfunction in obesity and diabetes models,^5^ ^,^ ^6^ attenuates metabolic-associated fatty liver disease,^7^ and exerts anti-inflammatory effects in experimental colitis.^8^ ^,^ ^9^ Collectively, these findings suggest that *B. uniformis* plays an important role in maintaining host metabolic and immune homeostasis. Given that metabolic dysregulation and intestinal barrier dysfunction are key features of ALD, *B. uniformis* may represent a promising microbial candidate for therapeutic intervention. However, whether *B. uniformis* is altered during ALD progression and whether it directly influences alcohol-induced liver injury remain largely unknown.

Accumulating evidence indicates that microbial metabolites are critical mediators of gut–liver communication.^10^ Rather than acting solely through bacterial colonization, specific gut microbes frequently exert systemic effects through the production of bioactive metabolites capable of regulating host metabolism and immunity.^11-13^ Several microbiota-derived metabolites have been reported to influence ALD progression by modulating hepatic lipid metabolism, inflammatory responses, and oxidative stress.^14^ In parallel, mitochondrial dysfunction is increasingly recognized as a hallmark of ALD and a major contributor to hepatocellular injury.^15^ Nevertheless, whether *B. uniformis* produces functional metabolites capable of regulating hepatic mitochondrial homeostasis during ALD has not been investigated.

In the present study, we found that *B. uniformis* is depleted in patients with ALD and that supplementation with live *B. uniformis* alleviates alcohol-induced liver injury. Mechanistically, this protective effect involves *B. uniformis*-derived ornithine, which contributes to hepatic mitochondrial homeostasis via Sirt4. Furthermore, dietary flaxseed oil may enhance this pathway by promoting *B. uniformis* abundance. These findings reveal a previously unrecognized *B. uniformis*–ornithine–Sirt4 axis that may serve as a potential target for therapeutic intervention in ALD.

## 2. Materials and methods

### 2.1. Human studies

The 16S rDNA gene amplicon sequencing dataset for the ALD population (accession number: CRA037460) was obtained from the GSA-Human database (Genome Sequence Archive - Human) hosted by the National Genomics Data Center (CNCB), China National Center for Bioinformation (https://ngdc.cncb.ac.cn/gsa-human).

### 2.2. Animal experiments

Wild-type C57BL/6J mice were purchased from GemPharmatech Co., Ltd. (Nanjing, China) and maintained in a specific pathogen-free (SPF) facility at the Experimental Animal Center of Anhui Medical University. All experimental procedures were approved by the Institutional Animal Care and Use Committee of Anhui Medical University and conducted in accordance with the relevant ethical guidelines (approval number: No. LLSC20241969). The individual mouse was the experimental unit for the in vivo experiments. Mice were randomly allocated to the experimental groups. Mice were monitored daily, and no mortality or adverse events occurred; CO₂ euthanasia was performed at the scheduled endpoint.

### 2.3. NIAAA model of ALD and treatments

Female C57BL/6J mice (10–12 weeks old) were used. The NIAAA (Gao-Binge) model was established as illustrated in the figures. Pair-fed (PF) mice received a Lieber-DeCarli liquid diet for 15 days, while ethanol-fed (EtOH) mice were fed a stepwise increasing ethanol diet (1%, 3%, 5%) for 5 days, followed by 5% ethanol from days 6 to 15. On day 16, EtOH mice received a single oral gavage of ethanol (5 g/kg body weight), and PF mice received an isocaloric dose of dextrin maltose. Nine hours post-gavage, mice were euthanized by CO₂ inhalation at a chamber volume displacement rate of 30% per minute before collection of blood, liver, intestines, and other tissues.

For bacterial supplementation, mice received oral gavage of live *B. uniformis* (4 × 10^8^ CFU/200 μL PBS) every other day. Heat-killed *B. uniformis* was administered at the same dose for the EtOH + heat-killed group. PF and EtOH groups received PBS as a control. Ornithine (KM19572, KKL MED, USA) was dissolved in PBS and gavaged at 400 mg/kg every other day. The Sirt4 inhibitor SIRT4-IN-1 (HY-163316, MCE, Shanghai, China) was dissolved in DMSO and PBS and administered intraperitoneally at 5 mg/kg every other day. For dietary interventions, 5% flaxseed oil (8001-26-1, MACKLIN, Shanghai, China) was incorporated into the diet throughout the experiment.

To generate microbiota-depleted mice, the animals were provided ad libitum with sterile drinking water containing ampicillin (1 g/L), neomycin (1 g/L), metronidazole (1 g/L), and vancomycin (0.5 g/L) for 7 days before induction of the ALD model.

### 2.4. Bacterial culture and growth analysis

*B. uniformis* (139204, BNCC, Beijing, China) was cultured on Columbia blood agar under anaerobic conditions at 37 °C for 24 h. Bacterial growth was monitored by measuring optical density at 600 nm (OD600). Cells were harvested by centrifugation (3500 rpm, 5 min), resuspended in sterile anaerobic PBS, and used for experiments. For testing the effect of flaxseed oil, fresh cultures were inoculated into medium containing 0–10% flaxseed oil and grown anaerobically for 24 h, with OD600 measured every 6 h.

### 2.5. Cell culture and treatments

AML12 mouse hepatocytes or primary hepatocytes were cultured in DMEM/F12 or DMEM medium supplemented with 10% FBS and 1% penicillin-streptomycin at 37 °C with 5% CO₂. At 60–70% confluence, cells were treated with ethanol (200 mM) for 24 h, with or without 2 h pre-incubation with ornithine (5 mM). For Sirt4 inhibition, SIRT4-IN-1 (10 μM, KM19572, KKL MED, USA) was added 2 h before ornithine exposure. Treated cells were used for downstream assays including Oil Red O staining (IO1720, Solarbio).

### 2.6. Isolation of primary hepatocytes

Primary hepatocytes were isolated from 6–8-week-old female C57BL/6J mice via portal vein perfusion with EGTA/glucose solution (Solution I) for 10 min, followed by collagenase type I/DNase I/glucose solution (Solution II) for 15 min. The liver was excised, filtered through a 70 μm mesh, and cells were washed by centrifugation (500 rpm, 4 °C, three times; then 700 rpm for 5 min). Cells were seeded in 6-well plates at 200 μL per well.

### 2.7. Histopathology and staining

Liver tissues were fixed in 4% paraformaldehyde, embedded in paraffin, sectioned at 4 μm, and subjected to H&E and MPO (ab208670, Abcam, Cambridge, UK) staining. Cryosections (10 μm) were stained with Oil Red O for lipid detection.

### 2.8. Immunofluorescence

Intestinal tissues were fixed, paraffin-embedded, sectioned at 5 μm, and stained with anti-Occludin (DF7504, Affinity Biosciences, USA) and anti-ZO-1 (21773-1-AP, Proteintech, China) following standard protocols.

### 2.9. Ornithine quantification

Serum, liver homogenates, and bacterial culture supernatants were analyzed using the ornithine ELISA kit (SPS-22049, SAIPEISEN Biotechnology, China) according to the manufacturer’s instructions, with absorbance measured at 450 nm.

### 2.10. NAD+/NADH detection

Mouse liver tissue (20−30 mg) was homogenized in pre-chilled extraction buffer (200 μL/10 mg tissue) and centrifuged. The supernatant was collected; one aliquot was heated at 60 °C for 30 min to decompose NAD⁺ (for NADH measurement), while the other was kept for total NAD⁺/NADH measurement. Samples (20 μL) or NADH standards were mixed with 90 μL ethanol dehydrogenase and 40 μL WST-8 solution from the Enhanced NAD⁺/NADH Assay Kit (S0176S, Beyotime, China), incubated at 37 °C for 30-45 min in the dark, and absorbance was read at 450 nm. Total NAD⁺/NADH and NADH concentrations were calculated from the standard curve, and NAD⁺ concentration was derived by subtraction.

### 2.11. Biochemical assays, serum ethanol and LPS detection

Serum ALT and AST were measured using Mindray automated analyzers with commercial kits (ALT: V501534; AST: V257024, China). Liver tissue TG, MDA, GSH, and T-SOD were quantified using kits from Nanjing Jiancheng Bioengineering Institute, normalized to protein content. Ethanol concentrations in freshly collected mouse serum were promptly measured using an Ethanol Assay Kit with WST-8 (S0240S; Beyotime, China) according to the manufacturer’s instructions. Serum LPS concentrations were measured by ELISA using a Mouse LPS ELISA Kit (ELK2993; ELK Biotechnology, China) according to the manufacturer’s instructions.

### 2.12. Mitochondrial membrane potential assay

Mitochondrial membrane potential in AML12 cells was assessed using the Enhanced Mitochondrial Membrane Potential Assay Kit (JC‑1) (C2003S, Beyotime, China). Treated cells were incubated with JC‑1 working solution for 20 minutes in the dark. The ratio of red (aggregate) to green (monomer) fluorescence intensity was measured as an indicator of mitochondrial membrane potential, where a decreased ratio reflects mitochondrial depolarization. Fluorescence was detected using flow cytometry or fluorescence microscopy.

### 2.13. Mitochondrial superoxide levels detection

Mitochondrial superoxide levels were detected using the MitoSOX™ Red Kit (S0061S, Beyotime, China). Mouse primary hepatocytes or AML12 cells were seeded in culture plates, treated as indicated, and then incubated with MitoSOX™ Red working solution (final concentration 5 μM) at 37 °C for 10−30 min in the dark. The supernatant was discarded, and cells were washed 2-3 times with pre-warmed PBS, followed immediately by fluorescence intensity measurement using a fluorescence microscope.

### 2.14. Western blot

Liver tissues or cells were homogenized and lysed in RIPA buffer (P0013B, Beyotime, China) containing protease and phosphatase inhibitors (P1045, Beyotime, China). Protein concentration was determined using the Enhanced BCA Protein Assay Kit (P0010, Beyotime, China). Lysates were mixed with one-quarter volume of loading buffer and boiled at 100 °C for 7 min. Proteins were separated on 7.5% or 10% acrylamide gels and transferred onto PVDF membranes (IPVH00010, MERCK, Germany). Membranes were blocked with 5% non-fat milk or 5% BSA in TBST for 2 h, incubated with primary antibodies overnight, and then with HRP-conjugated secondary antibodies. Protein bands were visualized using the Super ECL Chemiluminescence Kit (P10100, NCM Biotech, China) and quantified with ImageJ. Primary antibodies used included Sirt4 (24662, CST, USA), OPA1 (66583‑1‑Ig, Proteintech, China), MFN1 (13798‑1‑AP, Proteintech, China), DRP1 (12957‑1‑AP, Proteintech, China), FIS1 (10956‑1‑AP, Proteintech, China), Cyp2e1 (67263‑1‑Ig, Proteintech, China), and β‑actin (66009‑1‑Ig, Proteintech, China).

### 2.15. qRT-PCR

Total RNA was extracted from 50 mg liver tissue using TRIGene reagent, reverse transcribed into cDNA, and analyzed using SYBR Green qPCR (Q711‑02, Vazyme, China). *β*-actin was used as the internal control, and relative expression was calculated by 2^−^ΔΔCt.

### 2.16. Transmission electron microscopy (TEM)

Fresh mouse liver tissue (~1 mm^3^) was immediately placed in electron microscopy fixative. Samples were fixed at 4 °C, rinsed three times with 0.1 M phosphate buffer (PBS, pH 7.4, 15 min each), and post-fixed with 1% osmium tetroxide in 0.1 M PBS for 2 h at room temperature in the dark. After three PBS washes, tissues were dehydrated through graded ethanol (30%–100%) and acetone series, then infiltrated stepwise with acetone–812 embedding resin mixtures (1:1, 1:2) and finally with pure 812 resin. Samples were embedded and polymerized at 60 °C for 48 h. Ultrathin sections (60–80 nm) were cut, mounted on 150-mesh copper grids, stained with 2% uranyl acetate in saturated alcoholic solution for 8 min in the dark, washed with 70% ethanol and ultrapure water, and examined under a transmission electron microscope. Images were acquired and analyzed accordingly.

### 2.17. Fluorescence in situ hybridization (FISH)

To detect *B. uniformis* in mouse intestinal tissues, a Cy3-labeled oligonucleotide probe specific to *B. uniformis* (5’-CY3- CTCAGAACCCCTATCCATCGACGGCTTGGTGGGCCGTTA -3’) was used. Fixed, paraffin-embedded tissue sections were deparaffinized and rehydrated, followed by treatment with proteinase K (20 μg/mL) at 37 °C for 30 min to facilitate permeabilization. Sections were then incubated with prehybridization buffer at 37 °C for 1 h, followed by overnight hybridization at 37 °C with the probe at a working concentration of 5ng/ul. After stringent washing to remove unbound probes, nuclei were counterstained with DAPI. Images were acquired using a fluorescence microscope, and *B. uniformis* signals were quantified as the number of FISH-positive spots per villus.

### 2.18. 16S rDNA sequencing

Microbial DNA from mouse intestinal contents was extracted using the HiPure Soil DNA Extraction Kit (Magen, Guangzhou, China). The V3–V4 region of the 16S rDNA gene was amplified with primers 341F (5′‑CCTACGGGNGGCWGCAG‑3′) and 806 R (5′‑GGACTACHVGGGTATCTAAT‑3′). PCR products were purified with P‑beads (Beckman, USA), quantified using a Qubit 3.0 fluorometer, and libraries were constructed using the Illumina DNA Prep Kit (Illumina, USA). Sequencing was performed on the NovaSeq 6000 platform (PE250). Data processing included quality filtering, chimera removal, operational taxonomic units (OTUs) clustering (Usearch). Taxonomic annotation was performed against the SILVA database (version 138.2), and taxonomy was classified using the RDP Classifier (version 2.2). Downstream analyzes were based on OTUs. Functional prediction of KEGG metabolic pathways was performed using PICRUSt2 (version 2.5.3). Diversity analyzes focused on *β*-diversity. LEfSe analysis (version 1.0) was performed to identify differentially abundant bacterial taxa between groups from the phylum to species levels, using an LDA score > 2 and *P* < 0.05 as thresholds.

### 2.19. Proteomics analysis

Mouse liver samples were homogenized in lysis buffer containing protease inhibitors. After protein extraction, quantification, and enzymatic digestion, peptides were desalted and analyzed using high-pH reversed-phase separation coupled with LC-MS/MS on an Orbitrap Fusion Lumos system integrated with an EASY-nLC 1200 (Thermo Fisher Scientific, USA). Raw data were processed using Spectronaut X (Bionosys AG) against the Uniprot database. Proteins with fold change >1.2 and *p* < 0.05 were considered differentially expressed and subjected to GO and KEGG functional enrichment analyzes. Statistical analyzes and visualization were performed using R software.

### 2.20. LC-MS nontargeted metabolomics

Mouse intestinal contents were rapidly frozen in liquid nitrogen. After sample preparation, metabolites were analyzed by LC-MS/MS (Vanquish UHPLC, Thermo Fisher Scientific, USA) using a Waters ACQUITY UPLC BEH Amide column (2.0 mm × 50 mm × 1.7 μm). Mass spectra were acquired on an Orbitrap Exploris 120 in both positive (POS) and negative (NEG) ion modes. Raw data were converted to mzXML (ProteoWizard), followed by peak picking, alignment, and integration. Pathway enrichment was performed using the KEGG database, with statistical analysis and visualization conducted in R.

### 2.21. Statistical analysis

Data are presented as mean ± SEM. Statistical analyzes were performed using GraphPad Prism 10.0. Comparisons between two groups were conducted using unpaired t-tests; multiple groups were analyzed by one-way ANOVA followed by Tukey’s post-hoc test. A *p*-value < 0.05 was considered statistically significant.

## 3. Results

### 3.1. *B. uniformis* abundance is reduced in ALD, whereas its supplementation alleviates alcohol-induced liver pathology in microbiota-depleted mice

To identify gut microbial alterations associated with alcohol-associated liver disease (ALD), we reanalyzed the gut microbiota dataset, which was previously reported in our published study,^16^ to identify bacterial species potentially associated with disease progression. Principal component analysis (PCA) revealed a clear separation between HC and ALD subjects, indicating substantial remodeling of the gut microbial community during disease progression (Figure 1a). Species-level differential abundance analysis identified *Bacteroides uniformis* as one of the most significantly depleted bacterial species in ALD patients (Figure 1b). Given the reported beneficial effects of *B. uniformis* in metabolic disorders, we hypothesized that its depletion may contribute to ALD pathogenesis.

**Figure 1.**
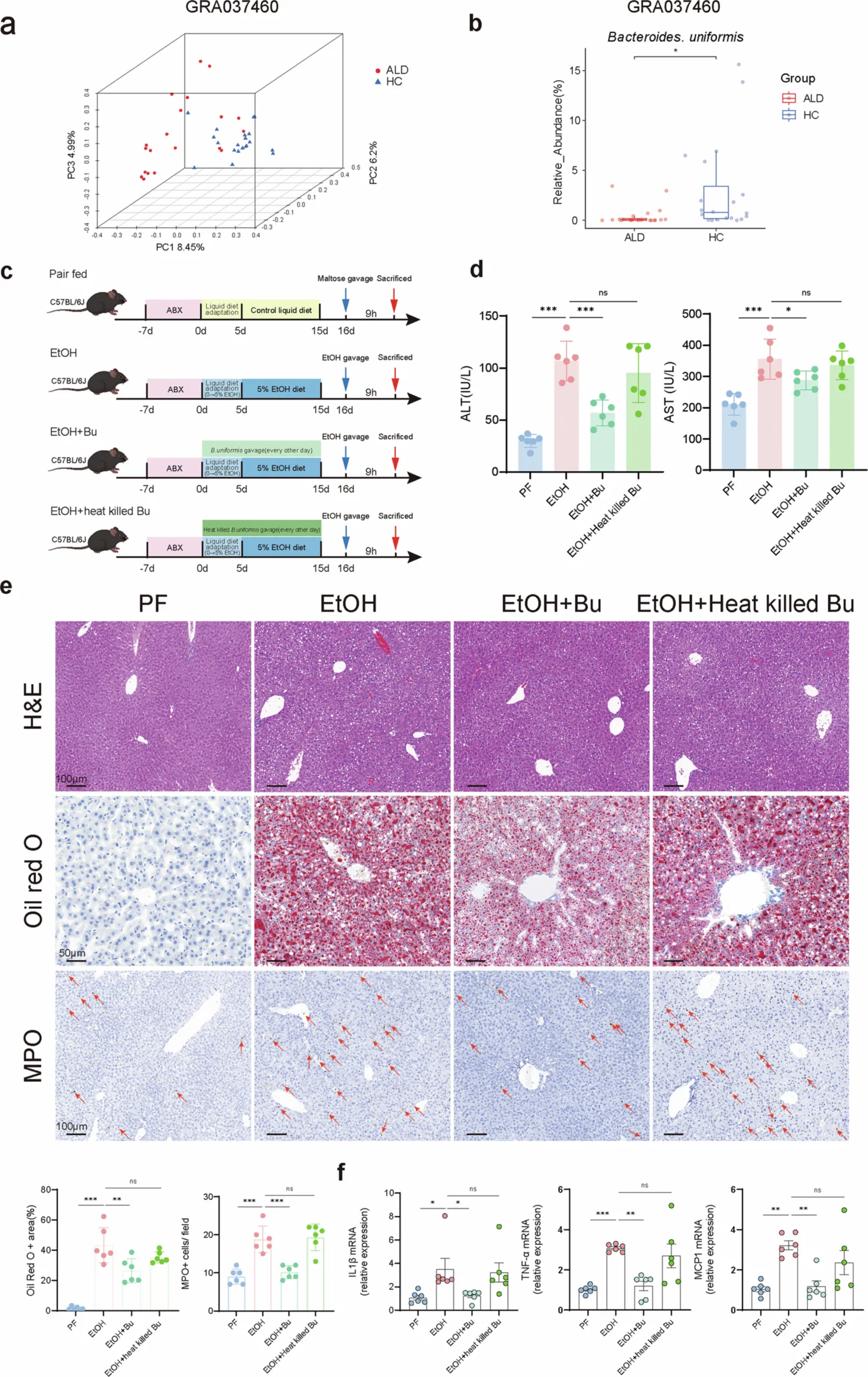
*B. uniformis* abundance is reduced in ALD, while its supplementation alleviates alcohol-induced liver pathologies in microbiota-depleted mice (a) PCoA 3D plot showing *β*-diversity of the gut microbiota between HC and ALD population (dataset CRA037460 from the GSA database) (*n* = 20 per group); (b) *B. uniformis* abundance in stool samples of HC and ALD population (*n* = 20 per group); (c) Experimental timeline illustrating the study design, including duration of antibiotic sustained cleansing, duration of pair fed or ethanol (EtOH) feeding, and duration and dosage of (heat-killed) *B. uniformis* gavage (*n* = 6 per group); (d) Serum ALT and AST level at the end of the feeding period (*n* = 6 per group); (e) Representative images of liver sections with H&E staining, Oil Red O staining, and MPO immunohistochemical staining, along with quantification of Oil Red O-positive area (*n* = 6 per group) and MPO-positive cell count per field (*n* = 6 per group); (f) Quantitated data of inflammatory genes IL-1β, TNF-*α*, and MCP1 in murine liver tissues detected by qPCR (*n* = 6 per group). PCoA, Principal Coordinate Analysis; HC, healthy controls; ALD, alcohol-associated liver disease; ALT, alanine aminotransferase; AST, aspartate aminotransferase; MPO, myeloperoxidase; PF, pair-fed; EtOH, ethanol-fed; EtOH + Bu, ethanol-fed with *B. uniformis* gavage; EtOH + heat killed Bu, ethanol-fed with heat killed *B. uniformis* gavage. Data was presented as mean ± SEM. *p < 0.05, **p < 0.01, ***p < 0.001. Significant differences between means were determined by t-test (for two groups) or by one-way ANOVA followed by Tukey's multiple comparisons test (for three or more groups).

To test whether *B. uniformis* directly protects against ALD, mice were pretreated with a broad-spectrum antibiotic cocktail for one week to deplete endogenous gut microbiota, thereby minimizing the influence of pre-existing bacteria. Following microbiota depletion, mice received either live or heat-killed *B. uniformis* throughout the NIAAA alcohol feeding protocol (Figure 1c). Live *B. uniformis* markedly attenuated ethanol-induced liver injury, as evidenced by reduced serum ALT and AST levels, whereas heat-killed bacteria failed to confer protection (Figure 1 d). Histological examination further demonstrated that live *B. uniformis* supplementation significantly alleviated hepatic steatosis and inflammatory cell infiltration (Figure 1e). Consistently, hepatic triglyceride accumulation was reduced (Fig. S1a), accompanied by normalization of genes involved in lipid metabolism and inflammatory responses (Figure 1f and Fig. S1b). In parallel, *B. uniformis* restored hepatic redox homeostasis, as reflected by reduced MDA levels and increased GSH and T-SOD activities (Fig. S1c). MPO staining further confirmed a marked reduction in hepatic neutrophil infiltration following *B. uniformis* supplementation (Figure 1e).

The observation that only live, but not heat-killed, *B. uniformis* conferred protection suggested that secreted bacterial metabolites, rather than structural bacterial components, mediate these effects. We therefore sought to identify the metabolites responsible for the hepatoprotective activity of *B. uniformis*.

### 3.2. *B. uniformis* colonization increases intestinal ornithine production in ALD mice

To verify successful engraftment following bacterial supplementation, we first characterized the intestinal microbiota of antibiotic-pretreated ALD mice. 16S rDNA sequencing confirmed a marked enrichment of *B. uniformis* in the EtOH + Bu group compared with ethanol-fed controls (Figure 2a−b, Fig. S2a). Consistent with this, *β*-diversity analysis revealed a distinct overall microbial community structure following *B. uniformis* supplementation (Figure 2c, Fig. S2b), and functional prediction analysis suggested enhanced amino acid and carbohydrate metabolic capacities in the supplemented group (Fig. S2c-d). Furthermore, intestinal FISH analysis revealed an increased number of *B. uniformis*-positive signals in the EtOH+Bu group, whereas heat-killed *B. uniformis* did not significantly alter these signals compared with the EtOH group (Figure 2d). These findings provide additional evidence that live *B. uniformis* administration increases the abundance of detectable *B. uniformis* signals in the intestine.

**Figure 2.**
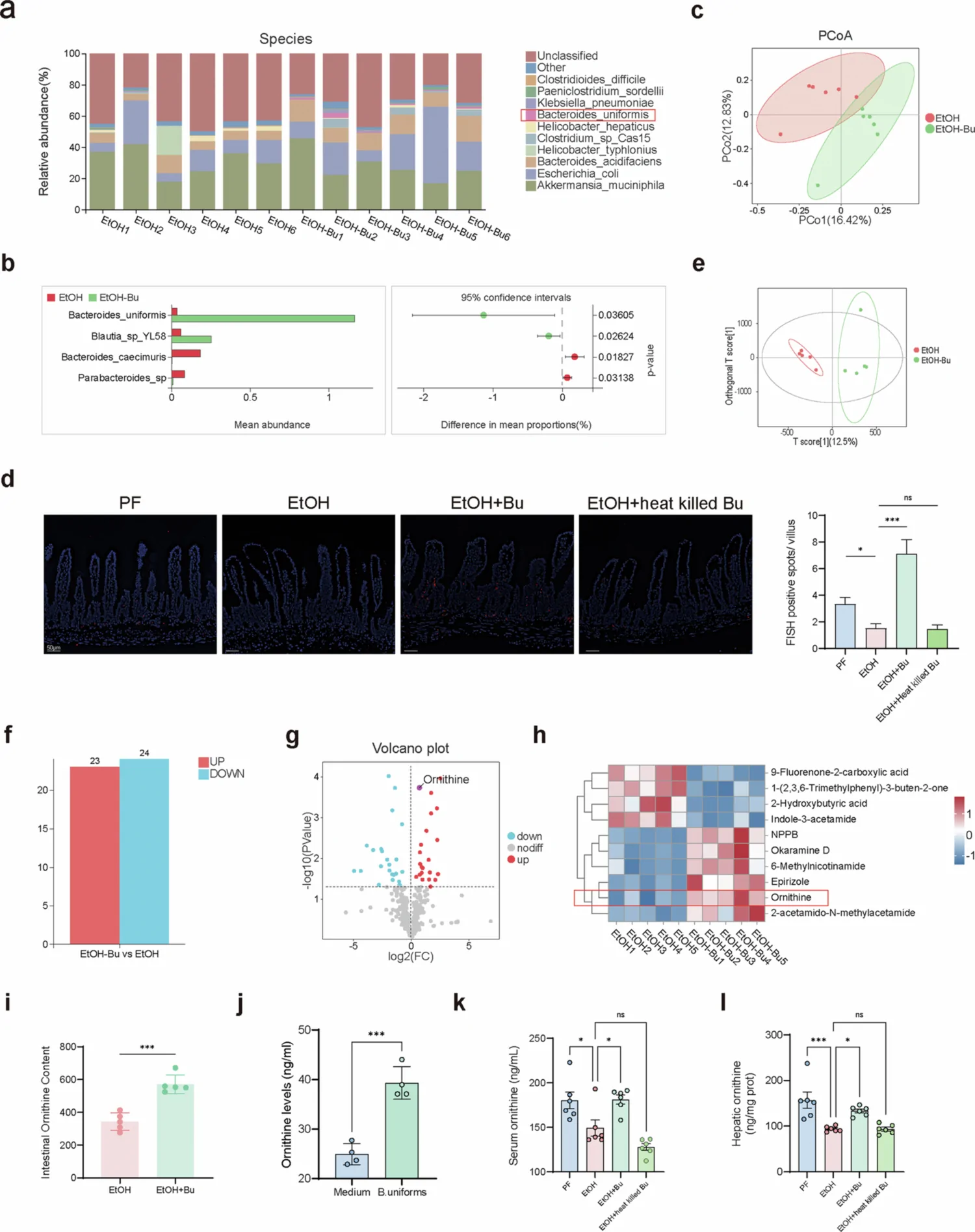
*B. uniformis* reshapes gut metabolism. (a) Relative abundance of gut microbiota at the species level for each sample (*n* = 6 per group); (b) Significance analysis of gut microbiota at the species level between two groups using Welch’s t test (*p* < 0.05) (*n* = 6 per group); (c) PCoA plot showing *β*-diversity of the gut microbiota (*n* = 6 per group); (d) Representative FISH staining of *B. uniformis* DNA (red) and nuclei (DAPI, blue) in intestine sections (*n* = 6 per group); (e-h) OPLS-DA plot (e), difference bar chart (f), volcano plot (g) and heatmap (h) of gut metabolites analyzed by LC (*n* = 5 per group); (i) Intestinal ornithine content detected by LC (*n* = 5 per group); (j) Ornithine level measured in blank medium or *B. uniformis* culture supernatants collected at 48 hours (*n* = 4 per group); (k) Serum ornithine level in mice at the end of the feeding period (*n* = 6 per group); (l) Hepatic ornithine level in mice at the end of the feeding period (*n* = 6 per group). PCoA, Principal Coordinate Analysis; OPLS-DA, Orthogonal Partial Least Squares Discriminant Analysis; LC, Liquid Chromatography; FISH, fluorescence in situ hybridization; PF, pair-fed; EtOH, ethanol-fed; EtOH + Bu, ethanol-fed with *B. uniformis* gavage; EtOH + heat killed Bu, ethanol-fed with heat killed *B. uniformis* gavage. Data was presented as mean ± SEM. *p < 0.05, **p < 0.01, ***p < 0.001. Significant differences between means were determined by t-test (for two groups) or by one-way ANOVA followed by Tukey's multiple comparisons test (for three or more groups).

We next investigated whether these microbial alterations were accompanied by changes in intestinal metabolites. Untargeted metabolomic profiling of intestinal contents showed a clear separation between the EtOH and EtOH+Bu groups in OPLS-DA (Figure 2e−f). Volcano plot analysis identified ornithine as one of the most significantly elevated metabolites following *B. uniformis* supplementation (Figure 2g−i). In vitro culture further confirmed that *B. uniformis* produces ornithine (Figure 2j), and in vivo, both serum and hepatic ornithine concentrations were restored in mice receiving live *B. uniformis*, whereas heat-killed *B. uniformis* had no significant effect (Figure 2k–l).

Together, these findings identify ornithine as a candidate effector metabolite downstream of *B. uniformis* and suggest a potential mechanistic link between bacterial colonization and host protection against ALD.

### 3.3. Ornithine alleviates disease progression in ALD mice

To determine whether ornithine mediates the hepatoprotective effects of *B. uniformis*, ALD mice were supplemented with ornithine throughout the NIAAA protocol (Figure 3a). Ornithine administration significantly attenuated ethanol-induced liver injury, as evidenced by reduced serum ALT and AST levels, decreased hepatic steatosis and triglyceride accumulation, and diminished inflammatory cell infiltration (Figure 3b−d). Hepatic expression of genes involved in lipid metabolism and inflammatory responses was similarly improved (Fig. S3a-b).

**Figure 3.**
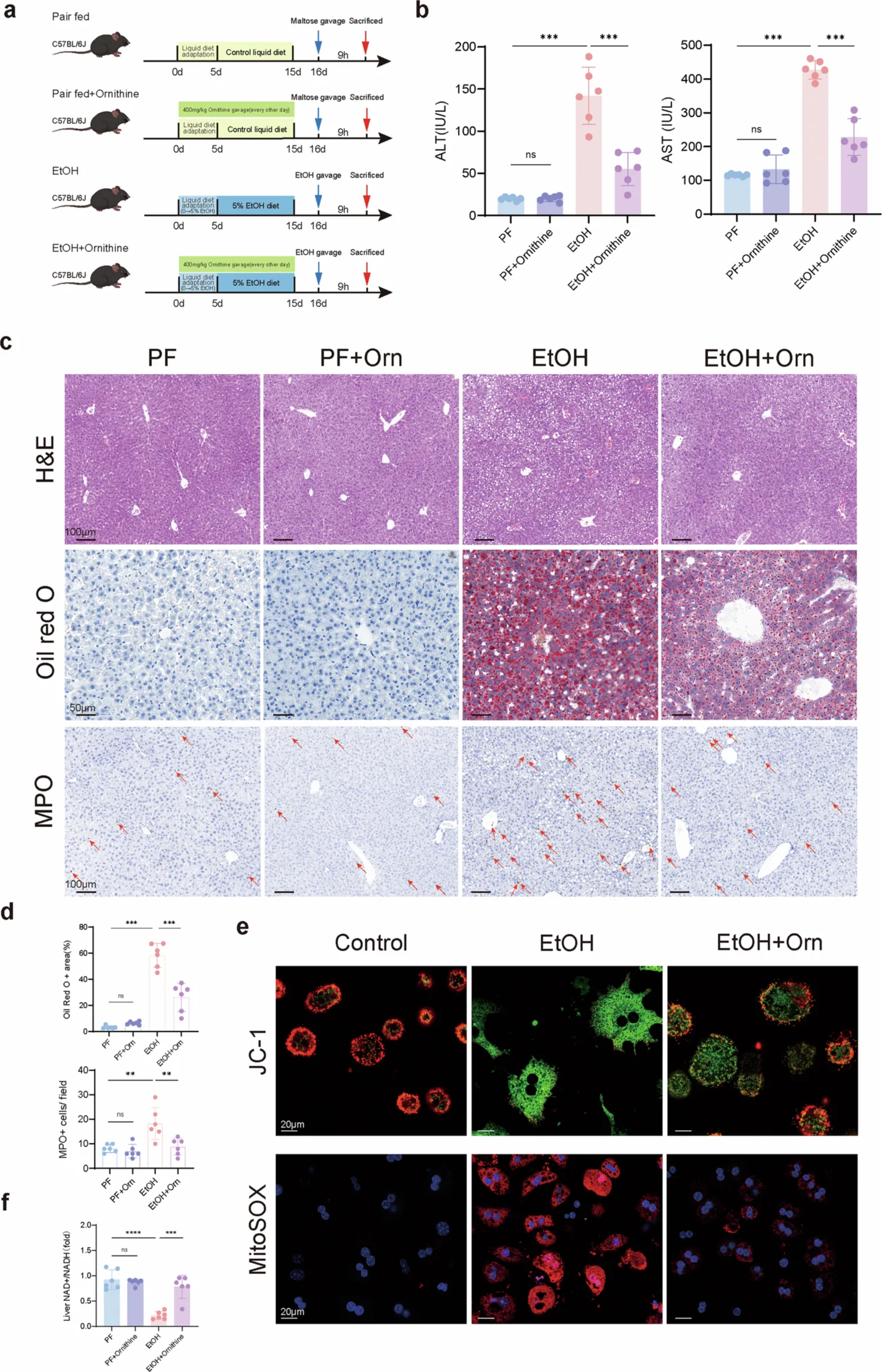
Ornithine improves disease progression in ALD mice. (a) Experimental timeline illustrating the study design, including duration of pair fed or ethanol (EtOH) feeding, and duration and dosage of ornithine gavage (*n* = 6 per group); (b) Serum ALT and AST level at the end of the feeding period (*n* = 6 per group); (c) Representative images of liver sections with H&E staining, Oil Red O staining and MPO immunohistochemical staining (*n* = 6 per group); (d) Quantification of Oil Red O-positive area and MPO-positive cell count per field in (c) (*n* = 6 per group); (e) Mitochondrial membrane potential with JC-1 stain and mitochondrial ROS generation with MitoSOX stain in primary mouse hepatocytes by immunofluorescence (*n* = 3 per group); (f) NAD + /NADH relative level measured in liver tissue homogenates (*n* = 6 per group). ALT, alanine aminotransferase; AST, aspartate aminotransferase; MPO, myeloperoxidase; MitoSOX, mitochondrial Superoxide; PF, pair-fed; PF + Orn, pair-fed with ornithine gavage; EtOH, ethanol-fed; EtOH + Orn, ethanol-fed with ornithine gavage. Data was presented as mean ± SEM. *p < 0.05, **p < 0.01, ***p < 0.001. Significant differences between means were determined by t-test (for two groups) or by one-way ANOVA followed by Tukey's multiple comparisons test (for three or more groups).

Given that mitochondrial dysfunction is a key pathogenic mechanism in ALD,^15^ we next examined whether ornithine influences mitochondrial function. In vitro, ornithine alleviated ethanol-induced mitochondrial membrane potential loss and reduced superoxide production (Figure 3e). As an intermediate metabolite in the urea cycle, ornithine may contribute to cellular redox balance by promoting NADH oxidation and thereby increasing the NAD⁺/NADH ratio. Since NAD⁺/NADH homeostasis is a central regulator of mitochondrial function, we further assessed this ratio in liver tissues and observed a significant increase following ornithine treatment (Figure 3f).

Notably, the overall protective phenotype induced by ornithine closely resembled that observed in *B. uniformis*-treated mice. Ornithine was well tolerated in pair-fed control mice, with no apparent adverse effects under physiological conditions. Collectively, these findings identify ornithine as a major functional mediator of *B. uniformis*-associated protection against ALD and prompted us to investigate its downstream hepatic targets.

### 3.4. Sirt4 mediates the protective effects of ornithine in ALD

To identify hepatic molecular targets underlying the protective effects of ornithine, we first performed quantitative proteomic analysis of liver tissues from ALD mice with or without *B. uniformis* supplementation. Principal component analysis revealed a clear separation between EtOH and *B. uniformis*-treated groups, indicating extensive remodeling of the hepatic proteome following bacterial intervention (Figure 4a). Differential protein analysis further identified a panel of proteins significantly altered between these two groups (Figure 4b).

**Figure 4.**
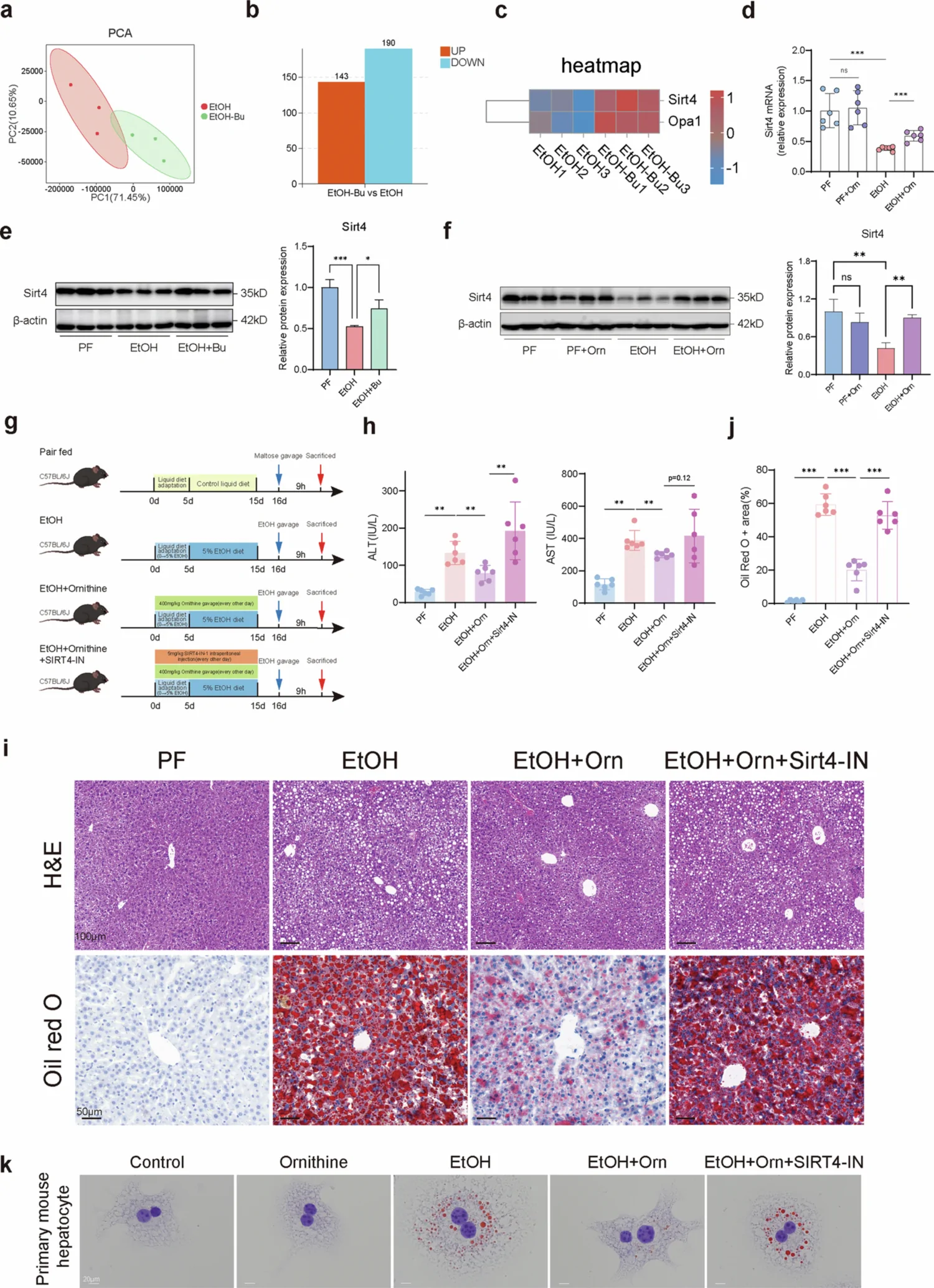
Sirt4 is the key hepatic target mediating the ameliorative effect of ornithine on ALD progression. (a) PCA plot showing *β*-diversity of global protein expression in mice liver by proteomics (*n* = 3 per group); (b) Difference bar graph of protein expression in mice liver by proteomics (*n* = 3 per group); (c) Heatmap show Sirt4 and Opa1 expression in each mouse liver by proteomics (*n* = 3 per group); (d) Quantitated data of Sirt4 mRNA in murine liver tissues detected by qPCR (*n* = 6 per group); (e-f) Western blot analysis of Sirt4 protein expression in liver tissues, with corresponding quantitative densitometry (*n* = 3 per group); (g) Experimental timeline illustrating the study design, including duration of pair fed or ethanol (EtOH) feeding, and duration and dosage of ornithine gavage and Sirt4 inhibitor (SIRT4-IN-1) intraperitoneal injection (*n* = 6 per group); (h) Serum ALT and AST level at the end of the feeding period (*n* = 6 per group); (i) Representative images of liver sections with H&E staining and Oil Red O staining (*n* = 6 per group); (j) Quantification of Oil Red O-positive area in (i); (k) Oil Red O staining of primary mouse hepatocytes (*n* = 3 per group). PCA, Principal Component Analysis; PF, pair-fed; EtOH, ethanol-fed; EtOH + Orn, ethanol-fed with ornithine gavage; EtOH + Orn + Sirt4-IN, ethanol-fed with ornithine gavage and Sirt4 inhibitor (SIRT4-IN-1) intraperitoneal injection. Data was presented as mean ± SEM. *p < 0.05, **p < 0.01, ***p < 0.001. Significant differences between means were determined by t-test (for two groups) or by one-way ANOVA followed by Tukey's multiple comparisons test (for three or more groups).

Among the differentially expressed proteins, Sirt4, a member of the NAD⁺-dependent sirtuin family, emerged as a key candidate due to its established role in mitochondrial metabolism and quality control.^17^ ^,^ ^18^ Proteomic data showed that Sirt4 was significantly increased in *B. uniformis*-treated mice compared with EtOH controls (Figure 4c). Notably, Opa1, a key regulator of mitochondrial fusion,^19^ ^,^ ^20^ exhibited a similar expression pattern, also being upregulated following *B. uniformis* supplementation (Figure 4c), suggesting coordinated restoration of mitochondrial homeostasis at the proteomic level.

To further validate these findings, we assessed hepatic Sirt4 protein expression in two independent experiments. *B. uniformis* supplementation was evaluated in PF, EtOH, and EtOH+Bu groups (Figure 4e), while ornithine supplementation was evaluated in PF, PF+Orn, EtOH, and EtOH+Orn groups (Figure 4f). In both experiments, Western blot analysis showed that ethanol feeding significantly reduced Sirt4 protein levels, which were restored by the respective treatments. In vitro, ornithine also attenuated the ethanol-induced reduction in hepatocellular Sirt4 protein expression (Fig. S4a). Consistently, Sirt4 mRNA levels showed a similar trend (Figure 4d), confirming transcriptional regulation.

Together with proteomic data, these results support Sirt4 as a key metabolic node responsive to both ethanol-induced injury and microbial/metabolite intervention.

To determine the functional relevance of Sirt4, we performed pharmacological inhibition experiments. SIRT4-IN-1 treatment alone aggravated ethanol-induced liver injury and steatosis (Fig. S4b–e). In a separate animal experiment, SIRT4-IN-1 attenuated ornithine-mediated protection (Figure 4g–j), and it also reduced ornithine’s protective effect in hepatocytes (Figure 4k).

Collectively, these results identify Sirt4 as a critical hepatic mediator of ornithine action and provide a mechanistic link between microbial metabolites and mitochondrial metabolic regulation in ALD. These findings further suggest that Sirt4-dependent mitochondrial homeostasis represents a key downstream effector pathway of the *B. uniformis*–ornithine axis.

### 3.5. Ornithine preserves mitochondrial homeostasis through a Sirt4-dependent mechanism

Given the central role of Sirt4 in mitochondrial regulation, we next investigated whether restoration of mitochondrial homeostasis contributes to ornithine-mediated protection against ALD. To assess whether the protective effects were associated with altered ethanol exposure or CYP2E1 expression, we measured serum ethanol concentrations and hepatic CYP2E1 protein levels. Neither parameter differed significantly between the relevant treatment groups (Fig. S4f–i).

At the structural level, transmission electron microscopy revealed marked mitochondrial swelling and cristae disruption in hepatocytes from ethanol-fed mice. Ornithine supplementation largely preserved mitochondrial ultrastructure, whereas Sirt4 inhibition significantly attenuated this protective effect (Figure 5a).

**Figure 5.**
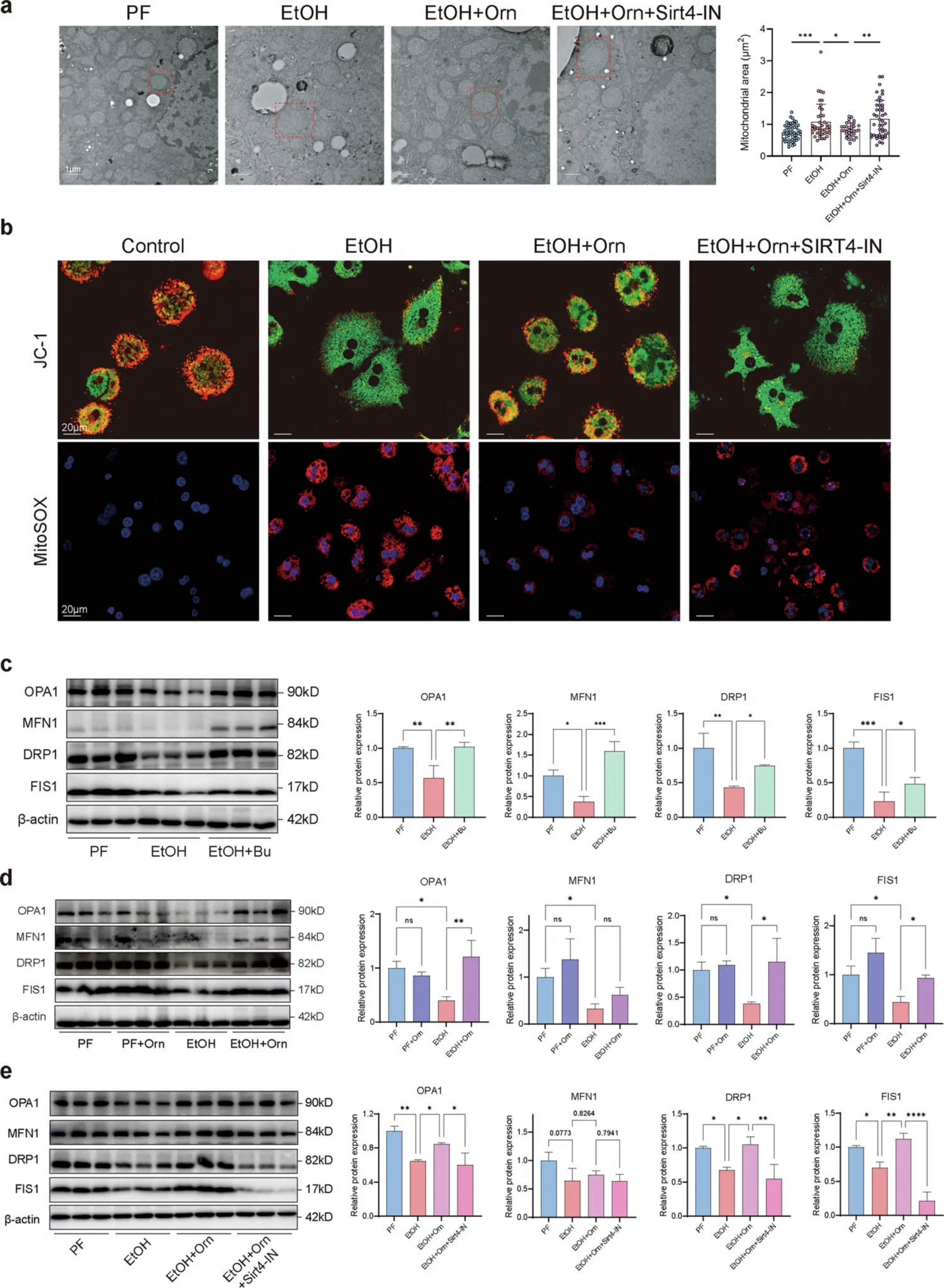
Hepatic Sirt4 upregulation ameliorates ALD through the regulation of mitochondrial dysfunction. (a) Mitochondrial morphology in mice liver tissues detected by electron microscopy, with the quantification (*n* = 3 per group); (b) Mitochondrial membrane potential with JC-1 stain and mitochondrial ROS generation with MitoSOX stain in primary mouse hepatocytes by immunofluorescence (*n* = 3 per group); (c-e) Western blot analysis of mitochondrial fusion and fission protein expression in liver tissues (*n* = 3 per group), with the quantification. MitoSOX, mitochondrial Superoxide; PF, pair-fed; PF + Orn, pair-fed with ornithine gavage; EtOH, ethanol-fed; EtOH + Bu, ethanol-fed with *B. uniformis* gavage; EtOH + Orn, ethanol-fed with ornithine gavage; EtOH + Orn + Sirt4-IN, ethanol-fed with ornithine gavage and Sirt4 inhibitor (SIRT4-IN-1) intraperitoneal injection. Data was presented as mean ± SEM. *p < 0.05, **p < 0.01, ***p < 0.001. Significant differences between means were determined by t-test (for two groups) or by one-way ANOVA followed by Tukey's multiple comparisons test (for three or more groups).

At the functional level, JC-1 staining demonstrated that ornithine restored ethanol-induced mitochondrial membrane potential loss in AML12 cells and primary hepatocytes, which was partially reversed by Sirt4 inhibition (Figure 5b and Fig. S4j). Consistently, MitoSOX staining indicated reduced mitochondrial oxidative stress following ornithine treatment, an effect weakened by Sirt4 inhibition (Figure 5b).

At the molecular level, ethanol exposure led to coordinated downregulation of key proteins involved in mitochondrial dynamics, including Opa1, Drp1, and Fis1, whereas both *B. uniformis* and ornithine supplementation restored their expression (Figure 5c–d), suggesting recovery of mitochondrial dynamic balance rather than unidirectional activation of fusion or fission pathways. MFN1 showed only minor changes, consistent with previous reports in the NIAAA model.^21^

Importantly, pharmacological inhibition of Sirt4 largely abolished the beneficial effects of ornithine on mitochondrial morphology, membrane potential, and oxidative stress (Figure 5a–b, 5e), indicating that Sirt4 is required for ornithine-mediated mitochondrial homeostasis.

Collectively, these findings demonstrate that ornithine preserves mitochondrial structural integrity and functional stability through a Sirt4-dependent mechanism, thereby contributing to protection against alcohol-induced hepatic injury.

### 3.6. Dietary flaxseed oil is associated with activation of the *B. uniformis*–Sirt4 pathway and amelioration of ALD

Having identified the *B. uniformis*–ornithine–Sirt4 pathway as a protective mechanism against ALD, we next explored whether this axis could be enhanced through dietary intervention. Previous studies have reported that polyunsaturated fatty acid-rich oils promote the growth of Bacteroides species.^22^ ^,^ ^23^ Consistent with these observations, in vitro culture experiments demonstrated that flaxseed oil promoted the proliferation of *B. uniformis* in a dose-dependent manner (Fig. S5a).

We therefore evaluated the effects of flaxseed oil supplementation in the NIAAA model (Figure 6a). Flaxseed oil significantly reduced serum ALT and AST levels and attenuated hepatic steatosis and lipid accumulation compared with ethanol-fed controls (Figure 6b−c).

**Figure 6.**
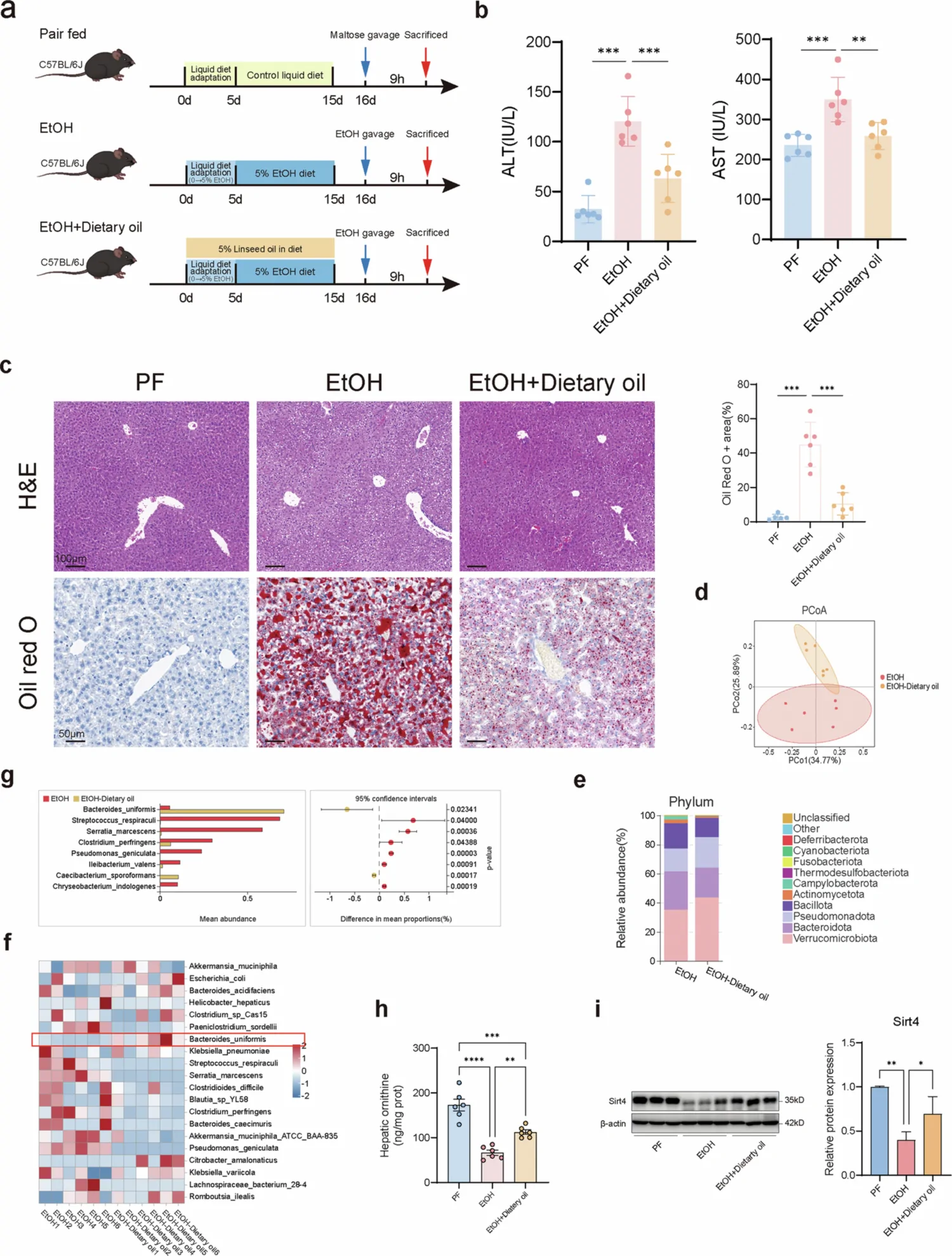
Dietary oil alleviates ALD in mice. (a) Experimental timeline illustrating the study design, including duration of pair fed or ethanol (EtOH) feeding, and duration and dosage of dietary oil (linseed oil) in diet (*n* = 6 per group); (b) Serum ALT and AST level at the end of the feeding period (*n* = 6 per group); (c) Representative images of liver sections with H&E staining and Oil Red O staining (*n* = 6 per group), along with quantification of Oil Red O-positive area (*n* = 6 per group); (d) PCoA plot showing *β*-diversity of the gut microbiota (*n* = 6 per group); (e) Relative abundance of gut microbiota at the phylum level (*n* = 6 per group); (f) Heatmap of gut microbiota at species level (*n* = 6 per group); (g) Significance analysis of gut microbiota at the species level between two groups using Welch’s t test (*p* < 0.05) (*n* = 6 per group); (h) Hepatic ornithine level in mice at the end of the feeding period (*n* = 6 per group); (i) Western blot analysis of Sirt4 protein expression in liver tissues, with corresponding quantitative densitometry (*n* = 3 per group). PCoA, Principal Coordinate Analysis; PF, pair-fed; EtOH, ethanol-fed; EtOH + Dietary oil, ethanol-fed with linseed oil in diet. Data was presented as mean ± SEM. *p < 0.05, **p < 0.01, ***p < 0.001. Significant differences between means were determined by t-test (for two groups) or by one-way ANOVA followed by Tukey's multiple comparisons test (for three or more groups).

To investigate whether these beneficial effects were associated with alterations in the gut microbiota, we performed 16S rDNA sequencing. *β*-diversity analysis revealed a distinct microbial community structure following flaxseed oil supplementation (Figure 6d−e, Fig. S5b). Notably, the relative abundance of *B. uniformis* was significantly increased in flaxseed oil-treated mice (Figure 6f−g, Fig. S5c), along with the increased hepatic ornithine levels (Figure 6h). Consistent with activation of the proposed pathway, hepatic Sirt4 expression was also restored following flaxseed oil supplementation (Figure 6i). Under ABX treatment, flaxseed oil did not significantly reduce serum ALT or AST levels but still decreased hepatic lipid accumulation, with a less pronounced improvement than that observed without ABX (Fig. S5d–f). These findings suggest that the gut microbiota contributes to the protective effects of flaxseed oil against ALD, while part of its effect on hepatic steatosis may persist independently of an intact microbiota.

Together, these findings suggest that flaxseed oil supplementation is associated with increased *B. uniformis* abundance and hepatic Sirt4 expression, highlighting a potentially translatable nutritional strategy for targeting the gut–liver axis in ALD.

## 4. Discussion

Our study provides the first evidence that *Bacteroides uniformis* produces ornithine, which targets hepatic Sirt4 to restore mitochondrial homeostasis and alleviate alcohol-associated liver disease (ALD). We further show that dietary oil supplementation can enrich *B. uniformis* and potentiate this protective effect (Figure 7).

**Figure 7.**
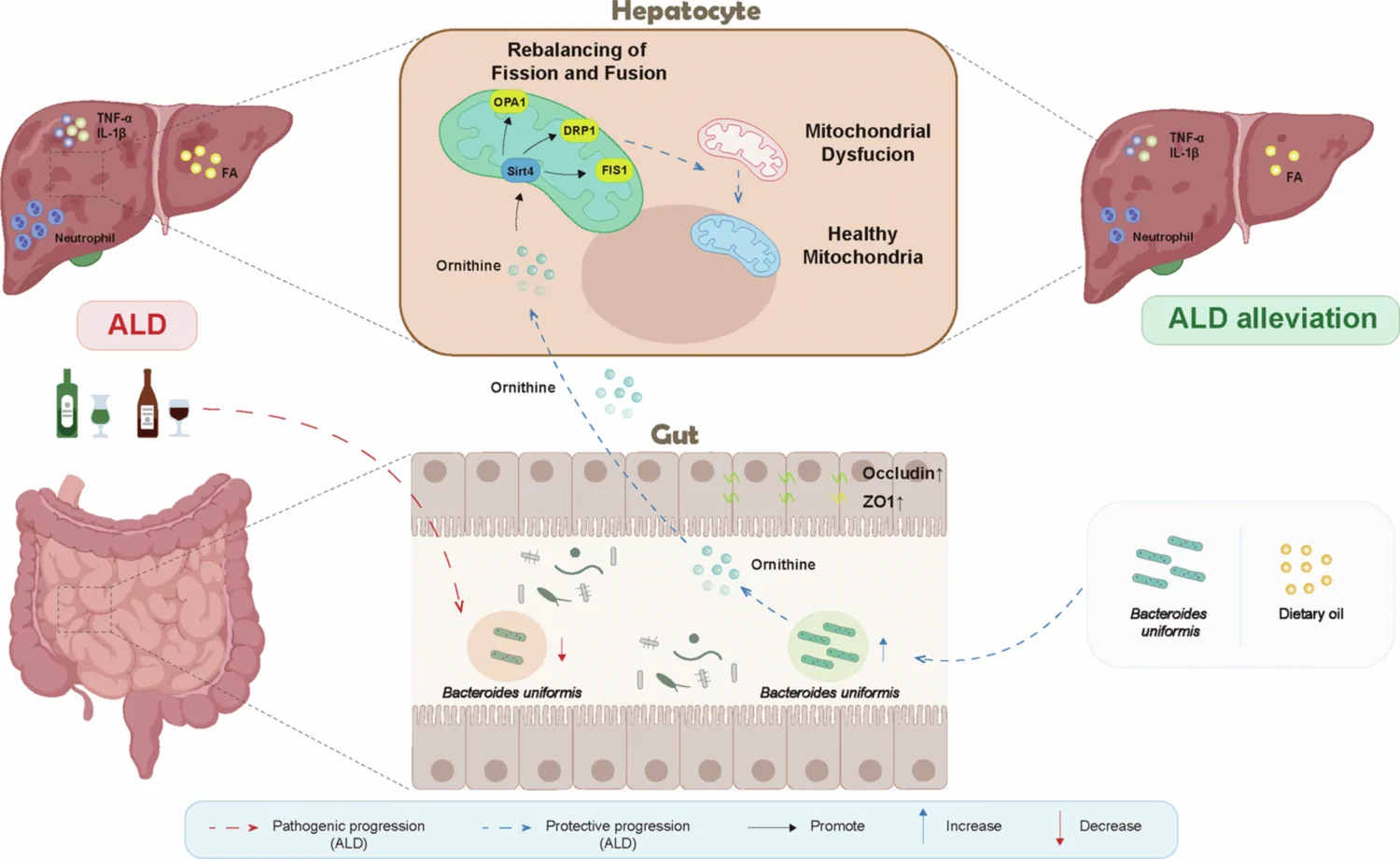
A schematic diagram of mechanism action. In alcohol‑associated liver disease (ALD), gut microbiota dysbiosis disrupts hepatic mitochondrial homeostasis. *Bacteroides uniformis* colonization in the murine gut alleviates ALD by secreting ornithine, which, through the gut‑liver axis, upregulates hepatic Sirt4 and regulates mitochondrial fusion/fission proteins (OPA1, DRP1, FIS1), ultimately restoring mitochondrial integrity. Notably, dietary oil intake enriches the abundance of *B. uniformis*, thereby restoring hepatic ornithine and Sirt4 levels and conferring therapeutic benefits.

Alcohol‑induced gut dysbiosis is a key driver of ALD progression.^24^ Previous studies have mainly focused on how alcohol promotes the overgrowth of pathobionts such as *Enterobacteriaceae* and reduces traditional probiotics like *Bifidobacterium* and *Lactobacillus,* ^25^ ^,^ ^26^ whereas the role of Bacteroides genus in ALD has received relatively little attention. *Bacteroides uniformis*, a Bacteroides species with high abundance in the human gut, has demonstrated protective potential in metabolic diseases such as obesity, insulin resistance, and non‑alcoholic fatty liver disease.^5^ ^,^ ^27^ ^,^ ^28^ Our findings complement previous studies showing that *B. uniformis* alleviates metabolic fatty liver by producing hexadecanedioic acid to inhibit the IRE1α‑XBP1s pathway, together revealing the metabolic plasticity of this species to adapt to different pathological environments through distinct metabolites.^7^

We analyzed gut contents by metabolomics and identified ornithine as one of the most elevated metabolites in ALD mice after *B. uniformis* supplementation. Ornithine is a vital precursor in polyamine synthesis and plays a critical role in regulating intestinal epithelial cell proliferation, differentiation, and tight junction protein expression.^29^ ^,^ ^30^ Direct administration of ornithine largely recapitulated the hepatoprotective effects of live bacteria in ALD mice, identifying ornithine as a key functional metabolite by which *B. uniformis* ameliorates ALD. Although not the primary focus of this study, we found that *B. uniformis* and ornithine improved the expression of intestinal tight junction proteins (Fig. S6b–c). In addition, live *B. uniformis* attenuated the ethanol-induced increase in serum LPS, whereas heat-killed *B. uniformis* did not (Fig. S6a). These findings suggest that restoration of epithelial homeostasis may represent an auxiliary mechanism contributing to the overall protective effects of the *B. uniformis*-ornithine axis. Notably, improvement in intestinal barrier function not only reduces endotoxin translocation but may also indirectly alleviate hepatic inflammation and lipid accumulation.^31^

Ornithine also provided mechanistic clues for hepatic protection. Our proteomic analysis highlighted Sirt4 as a key mitochondrial protein significantly altered by ethanol and restored by *B. uniformis* or ornithine. Sirt4, localized in the mitochondrial matrix with ADP-ribosyltransferase activity, regulates energy metabolism via deacetylation of enzymes such as glutamate dehydrogenase and pyruvate dehydrogenase.^32^ Recent studies have revealed its roles in hepatic lipid metabolism and mitochondrial quality control.^33^ ^,^ ^34^ Pharmacological inhibition of Sirt4 abolished the hepatoprotective effects of ornithine on liver enzymes, lipid deposition, and mitochondrial morphology, confirming that its protective action depends on Sirt4 function.

Mitochondrial dysfunction is a core event in ALD pathogenesis. Chronic alcohol exposure leads to mitochondrial protein damage, reduced respiratory chain complex activity, and impaired dynamics.^35^ The balance between fusion and fission is regulated by Opa1, MFN1/2, DRP1, and FIS1,^36^ and ALD is characterized by swollen mitochondria and decreased expression of these proteins.^21^ In our study, ornithine restored mitochondrial morphology and dynamics in a Sirt4-dependent manner, rescuing the ethanol-induced decline in membrane potential. Previous studies suggest that Sirt4 interacts with Opa1 and may influence cristae remodeling and mitochondrial fusion.^19^ ^,^ ^20^ ^,^ ^37^ Our data further validate that Sirt4 restores the expression of fission proteins Drp1 and Fis1, elucidating a key pathway by which ornithine corrects mitochondrial dysregulation in ALD. Mechanistically, SIRT4 is an NAD⁺-dependent mitochondrial deacylase with established lipoamidase activity.^38^ ^,^ ^39^ Previous studies have linked SIRT4 to OPA1-associated mitochondrial fusion and ERK–DRP1 signaling,^37^ ^,^ ^40^ suggesting that it may regulate mitochondrial dynamics through both fusion- and fission-related pathways. In our study, SIRT4 upregulation restored OPA1, DRP1, and FIS1 expression in ALD. Whether these effects involve direct deacylation of these proteins or indirect regulation remains unclear.

In addition to supplementation with probiotics or metabolites, modulating the gut microbiota through dietary strategies represents a clinically feasible intervention. Oils rich in ω‑3 polyunsaturated fatty acids can increase gut *Bacteroides abundance.* ^22^ ^,^ ^23^ Dietary oil increased intestinal *B. uniformis* abundance, upregulated hepatic Sirt4 expression, and improved ALD outcomes, supporting the development of “prebiotic‑based” nutritional strategies.^41^

Several limitations should be acknowledged. First, although ornithine is identified as a major mediator of *B. uniformis*-associated protection, additional bacterial metabolites may also contribute. Second, the requirement for Sirt4 was evaluated using pharmacological inhibition rather than genetic deletion; hepatocyte-specific Sirt4-deficient mice would provide stronger mechanistic evidence. Third, validation of the *B. uniformis*–ornithine–Sirt4 axis in human ALD cohorts is needed to support clinical translation.

In summary, our study identifies a gut–liver axis in which intestinal *B. uniformis* and its metabolite ornithine promote hepatic mitochondrial homeostasis via Sirt4, thereby protecting against alcohol-induced liver injury. Restoration of this signaling axis through probiotics, metabolite supplementation, or dietary interventions may represent a precision strategy for ALD prevention and treatment.

## Supplementary Material

Supplementary Material Supplementary Material

Supplementary Material Supplementary Material

## Data Availability

The proteomics and metabolomics reported in this paper have been deposited in the OMIX, China National Center for Bioinformation/Beijing Institute of Genomics, Chinese Academy of Sciences (https://ngdc.cncb.ac.cn/omix: accession no. OMIX018380 and no. OMIX018384). The 16 s rDNA data reported in this paper have been deposited in the Genome Sequence Archive (Genomics, Proteomics & Bioinformatics 2025) in National Genomics Data Center (Nucleic Acids Res 2026), China National Center for Bioinformation/Beijing Institute of Genomics, Chinese Academy of Sciences (GSA: CRA045923 and CRA045926) that are publicly accessible at https://ngdc.cncb.ac.cn/gsa.
