# Supplementary material for: Bacteroides uniformis-generated ornithine alleviates alcohol-associated liver disease via Sirt4-mediated mitochondrial regulation
Source: Gut Microbes. 2026 Nov 8;18(1):2756713. doi: 10.1080/19490976.2026.2756713 (PMC13757604; doi:10.1080/19490976.2026.2756713)
Supplement: Supplementary file 1 [file KGMI_A_2756713_SM4109.docx]

**Sup. Fig 1. Related to Figure 1.**

(a) TG levels measured in liver tissue homogenates (n=6 per group);

(b) Quantitated data of lipid metabolism genes in murine liver tissues detected by qPCR (n=6 per group);

(c) MDA, GSH and T-SOD levels in liver tissue homogenates (n=6 per group).

MDA, Malondialdehyde; GSH, Glutathione; T-SOD, Total Superoxide Dismutase; PF, pair-fed; EtOH, ethanol-fed; EtOH+Bu, ethanol-fed with *B. uniformis* gavage; EtOH+heat killed Bu, ethanol-fed with heat killed *B. uniformis* gavage.

Data was presented as mean ± SEM. *p < 0.05, **p < 0.01, **p < 0.001. Significant differences between means were determined by t-test (for two groups) or by one-way ANOVA followed by Tukey's multiple comparisons test (for three or more groups).

**Sup. Fig 2. Related to Figure 2**

(a) Cladogram showing significantly different taxa based on LEfSe analysis (n=6 per group)；

(b) PERMANOVA analysis of gut microbial β-diversity at the OTU level;

(c) Relative abundance of gut microbiota at the phylum, class, order, family, genus, species level (n=6 per group);

(d) Functional profile of the gut microbiota by PICRUSt2 analysis (n=6 per group).

LEfSe, Linear Discriminant Analysis Effect Size; PERMANOVA, Permutational Multivariate Analysis of Variance; PICRUSt2, Phylogenetic Investigation of Communities by Reconstruction of Unobserved States; PF, pair-fed; EtOH, ethanol-fed; EtOH+Bu, ethanol-fed with *B. uniformis* gavage; EtOH+heat killed Bu, ethanol-fed with heat killed *B. uniformis* gavage.

**Sup. Fig 3. Related to Figure 3**

(a) Quantitated data of inflammatory genes IL-1β, TNF-α and MCP1 in murine liver tissues detected by qPCR (n=6 per group);

(b) Quantitated data of lipid metabolism genes in murine liver tissues detected by qPCR (n=6 per group).

PF, pair-fed; PF+Orn, pair-fed with ornithine gavage; EtOH, ethanol-fed; EtOH+Orn, ethanol-fed with ornithine gavage.

**Sup. Fig 4. Related to Figure 4 and Figure 5**

(a) Western blot analysis of Sirt4 protein expression in AML12 cell, with the quantification (n=3 per group);

(b) Experimental timeline illustrating the study design, including duration of ethanol (EtOH) feeding, and duration and dosage of Sirt4 inhibitor (SIRT4-IN-1) intraperitoneal injection (n=5 per group);

(c) Serum ALT and AST level at the end of the feeding period (n=5 per group);

(d) Representative images of liver sections with H&E staining (n=5 per group);

(e) Representative images of liver sections with oil Red O staining, along with the quantification (n=5 per group);

(f-g) Serum ethanol concentration (n=6 per group);

(h-i) Western blot analysis of CYP2E1 protein expression in liver tissues (n=3 per group), with the quantification;

(j) Mitochondrial membrane potential in AML12 cells with JC-1 stain by immunofluorescence (n=3 per group).

PF, pair-fed; PF+Orn, pair-fed with ornithine gavage; EtOH, ethanol-fed; EtOH+Bu, ethanol-fed with *B. uniformis* gavage; EtOH+heat killed Bu, ethanol-fed with heat killed *B. uniformis* gavage; EtOH+Orn, ethanol-fed with ornithine gavage; EtOH+Sirt4-IN, ethanol-fed with Sirt4 inhibitor (SIRT4-IN-1) intraperitoneal injection.

Data was presented as mean ± SEM. *p < 0.05, **p < 0.01, **p < 0.001. Significant differences between means were determined by t-test (for two groups) or by one-way ANOVA followed by Tukey's multiple comparisons test (for three or more groups).

**Sup. Fig 5. Related to Figure 6**

(a) Growth curve (OD600) of *B. uniformis* under anaerobic conditions with varying dietary oil (Linseed oil) concentrations (n=3 per group);

(b) PERMANOVA analysis of gut microbial β-diversity at the OTU level;

(c) Cladogram showing significantly different taxa based on LEfSe analysis (n= 6 per group)；

(d) Experimental timeline illustrating the study design, including duration of antibiotic sustained cleansing, pair fed or ethanol (EtOH) feeding, and duration and dosage of dietary oil (linseed oil) in diet (n=6 per group)；

(e) Serum ALT and AST level at the end of the feeding period (n=6 per group);

(f) Representative images of liver sections with H&E staining and Oil red O staining, along with quantification of Oil Red O-positive area (n=6 per group).

LEfSe, Linear Discriminant Analysis Effect Size; PERMANOVA, Permutational Multivariate Analysis of Variance; PF, pair-fed; EtOH, ethanol-fed; EtOH+Dietary oil, ethanol-fed with linseed oil in diet; PF-abx, pair-fed with antibiotic sustained cleansing; EtOH-abx, ethanol-fed with antibiotic sustained cleansing; EtOH+Dietary oil-abx, ethanol-fed with linseed oil in diet with antibiotic sustained cleansing.

Data was presented as mean ± SEM. *p < 0.05, **p < 0.01, **p < 0.001. Significant differences between means were determined by t-test (for two groups) or by one-way ANOVA followed by Tukey's multiple comparisons test (for three or more groups).

**Sup. Fig6. *B. uniformis* or Ornithine alleviated intestinal barrier injury in ALD mice.**

(a) Serum LPS level (n=6 per group);

(b-c) Immunofluorescence of ileal tight junction proteins: Occludin (red), ZO-1 (green) and nuclei (DAPI, blue) (n=6 per group).

PF, pair-fed; EtOH, ethanol-fed; EtOH+Bu, ethanol-fed with *B. uniformis* gavage; EtOH+heat killed Bu, ethanol-fed with heat killed *B. uniformis* gavage; PF+Orn, pair-fed with ornithine gavage; EtOH+Orn, ethanol-fed with ornithine gavage.

Data was presented as mean ± SEM. *p < 0.05, **p < 0.01, **p < 0.001. Significant differences between means were determined by t-test (for two groups) or by one-way ANOVA followed by Tukey's multiple comparisons test (for three or more groups).
