# Supplementary material for: Bacteroides uniformis-generated ornithine alleviates alcohol-associated liver disease via Sirt4-mediated mitochondrial regulation
Source: Gut Microbes. 2026 Nov 8;18(1):2756713. doi: 10.1080/19490976.2026.2756713 (PMC13757604; doi:10.1080/19490976.2026.2756713)
Supplement: Supplementary file 2 [file KGMI_A_2756713_SM8077.docx]

Sup. Table 1 Primers used for RT-qPCR

| **Gene** | **Primer** | **Sequence (5’-3’)** | **Supplier** |
| --- | --- | --- | --- |
| Srebp-1c | Forward | GGAGCCATGGATTGCACATT | Tsingke Biotech |
|  | Reverse | GGCCCGGGAAGTCACTGT |  |
| Fasn | Forward | TGGTGAATTGTCTCCGAAAAGA | Tsingke Biotech |
|  | Reverse | CACGTTCATCACGAGGTCATG |  |
| CD36 | Forward | TTGTACCTATACTGTGGCTAAATGAGA | Tsingke Biotech |
|  | Reverse | CTTGTGTTTGAACATTTCTGCTT |  |
| Ppar-γ | Forward | CACAATGCCATCAGGTTTGG | Tsingke Biotech |
|  | Reverse | GCTGGTCGATATCACTGGAGATC |  |
| ACC1 | Forward | GGACAGACTGATCGCAGAGAAAG | Tsingke Biotech |
|  | Reverse | TGGAGAGCCCCACACACA |  |
| IL1β | Forward | TCGCTCAGGGTCACAAGAAA | Tsingke Biotech |
|  | Reverse | CATCAGAGGCAAGGAGGAAAAC |  |
| TNF-α | Forward | AGGCTGCCCCGACTACGT | Tsingke Biotech |
|  | Reverse | GACTTTCTCCTGGTATGAGATAGCAAA |  |
| Mcp-1 | Forward | CCAGCCTACTCATTGGGAT | Tsingke Biotech |
|  | Reverse | GGGCCTGCTGTTCACAGTT |  |
| Sirt4 | Forward | TAGCCGTTCACACGTAGCTG | Tsingke Biotech |
|  | Reverse | GGCCTGAAAGTCAATCCGCT |  |
| β-actin | Forward | CCTCTATGCCAACACAGT | Tsingke Biotech |
|  | Reverse | AGCCACCAATCCACACAG |  |
